# An Integrative Explainable Artificial Intelligence Approach to Analyze Fine-Scale Land-Cover and Land-Use Factors Associated with Spatial Distributions of Place of Residence of Reported Dengue Cases

**DOI:** 10.3390/tropicalmed8040238

**Published:** 2023-04-20

**Authors:** Hsiu Yang, Thi-Nhung Nguyen, Ting-Wu Chuang

**Affiliations:** 1 Department of Molecular Parasitology and Tropical Diseases, School of Medicine, College of Medicine, Taipei Medical University, Taipei 110, Taiwan; 2 International Ph.D. Program in Medicine, College of Medicine, Taipei Medical University, Taipei 110, Taiwan

**Keywords:** artificial intelligence, dengue fever, land cover land-use, EXtreme Gradient Boosting (XGBoost), Shapley Additive Explanation (SHAP)

## Abstract

Dengue fever is a prevalent mosquito-borne disease that burdens communities in subtropical and tropical regions. Dengue transmission is ecologically complex; several environmental conditions are critical for the spatial and temporal distribution of dengue. Interannual variability and spatial distribution of dengue transmission are well-studied; however, the effects of land cover and use are yet to be investigated. Therefore, we applied an explainable artificial intelligence (AI) approach to integrate the EXtreme Gradient Boosting and Shapley Additive Explanation (SHAP) methods to evaluate spatial patterns of the residences of reported dengue cases based on various fine-scale land-cover land-use types, Shannon’s diversity index, and household density in Kaohsiung City, Taiwan, between 2014 and 2015. We found that the proportions of general roads and residential areas play essential roles in dengue case residences with nonlinear patterns. Agriculture-related features were negatively associated with dengue incidence. Additionally, Shannon’s diversity index showed a U-shaped relationship with dengue infection, and SHAP dependence plots showed different relationships between various land-use types and dengue incidence. Finally, landscape-based prediction maps were generated from the best-fit model and highlighted high-risk zones within the metropolitan region. The explainable AI approach delineated precise associations between spatial patterns of the residences of dengue cases and diverse land-use characteristics. This information is beneficial for resource allocation and control strategy modification.

## 1. Introduction

Dengue fever (DF) is an important vectorborne disease prevalent worldwide. Two urban-dwelling mosquitoes, *Aedes aegypti* and *Aedes albopictus*, are the primary and secondary vectors for dengue virus transmission [1]. More than 129 countries with 390 million people are at risk of infection annually, of which 96 million dengue infection cases are symptomatic [2,3]. The clinical symptoms of dengue infection range from flulike syndromes to severe hemorrhage, plasma leakage, or organ impairment. Severe dengue infection is usually mediated by antibody-dependent enhancement, which develops after secondary infection with different dengue serotypes [4]. The rising incidence and expansion of geographical territory of dengue infection in tropical, subtropical, and temperate regions stems from global travel and environmental changes, resulting in a huge disease burden in the last two decades [5,6]. Vector control, source reduction, community education, and engagement are the mainstays for preventing dengue infection due to the lack of effective treatment. A licensed dengue vaccine (Dengvaxia) is recommended for people aged 9–45 years with a history of dengue infection [7]. However, this recommendation limits the number of eligible individuals. Therefore, preventing dengue transmission through nationwide vaccination programs in endemic countries is not recommended at the current stage.

Multiple factors affect global dengue transmission, including climate change/variation, land-use land-cover (LULC) types, sociodemographic characteristics, and international travel [2,6,8]. Climatic conditions are critical factors for determining interannual variations in dengue transmission. Therefore, many studies have attempted to use climatic factors to forecast the occurrence of dengue and establish an early warning system [9,10,11,12,13]. The influences of temperature, rainfall, and relative humidity on vector abundance and dengue transmission have been widely discussed [8,14,15]. In addition, regional climate phenomena derived from climate changes, like the El Niño Southern Oscillation (ENSO) or the Indian Ocean Dipole (IOD), have been linked to dengue transmission in many regions [16,17,18]. However, spatial patterns of dengue infection are more relevant to vector distributions, population density, and movement patterns of people, which are associated with various LULC characteristics. Climate-based models help design early warning systems for dengue to prevent potential outbreaks under extreme climatic conditions; however, spatial patterns driven by LULC characteristics are also important for defining intervention priority zones. Such a type of study is scarce in the literature and is worth further evaluation.

Recently, machine learning and artificial intelligence (AI) approaches to spatial epidemiology and mosquitoborne disease have started thriving [19,20]. Ensemble-tree-based machine learning approaches are useful for identifying important variables and predicting the spatial patterns of diseases. However, feature interpretation is equally important for epidemiological studies as the model’s performance [21]. Understanding precise relationships between environmental risk factors and dengue incidence could advance our knowledge of disease ecology to formulate appropriate dengue prevention and control policies. Therefore, recent approaches integrating machine learning and explainable AI have helped enhance feature interpretation using gradient-boosting models [22,23]. The availability of explainable approaches provides an excellent opportunity to evaluate different LULC characteristics and the spatial patterns of the residences of reported dengue cases.

Therefore, this study used the EXtreme Gradient Boosting (XGBoost) and Shapley Additive Explanation (SHAP) techniques to analyze multiple LULC type-related factors and spatial patterns of residences of reported dengue cases in Kaohsiung City (KC), Taiwan, which experienced unprecedented dengue outbreaks in 2014 and 2015 [17]. The availability of a fine-scale LULC survey released by the Taiwan government provides an excellent opportunity to deepen our understanding of the associations between landscape-level characteristics and spatial patterns of the residences of dengue cases using explainable AI approaches.

## 2. Materials and Methods

### 2.1. Study Area

**Figure 1:**
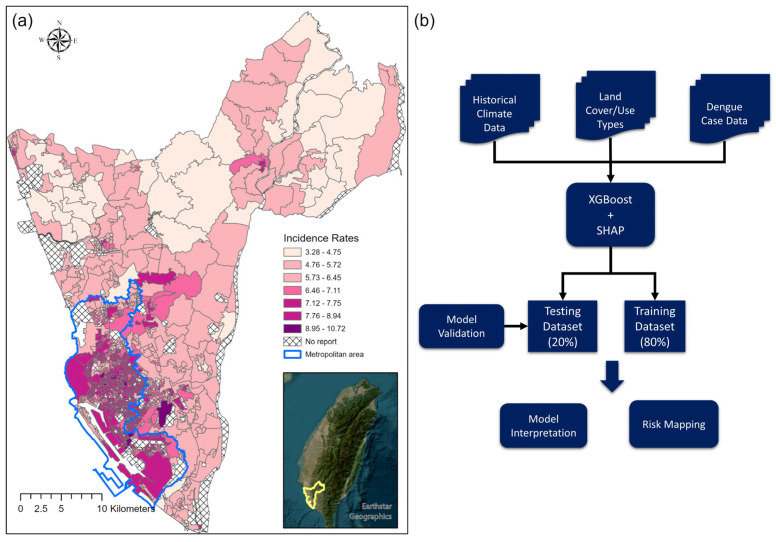
(**a**) Log-transformed dengue incidence rates in second-level dissemination areas in Kaohsiung City, Taiwan. (**b**) The schematic analytical flow of the modeling procedure.

KC is the second largest city in Taiwan, with 2.8 million inhabitants (population density: 9365 per square km; Figure 1a) [24]. It is the political and economic center of southern Taiwan (22°36′54″ N, 120°17′51″ E). The economic activities in KC mainly comprise industry, tourism, and agriculture. KC is approximately 100 km south of the Tropical of Cancer, with a mean temperature range of 19.7–29.4 °C and annual average precipitation of approximately 1968.2 mm. The primary rainy season is between May and September, and the “plum rains” (East Asian rainy season) in May and June bring continuous rainfall resulting in high humidity. The number of typhoons significantly determines precipitation fluctuations in July and August, affecting vector abundance and dengue transmission. Historically, KC is a dengue hotspot; more than 70% of dengue cases in Taiwan have been reported in the city [25]. Since 2000, 43,304 dengue cases have been reported in KC, with 34,782 reported in 2014 and 2015 [17]. Interactions between climate variations, the coexistence of *A. aegypti* and *A. albopictus* in urban and suburban regions and a high population density caused interannual outbreaks. Furthermore, continuous precipitation after the petrochemical gas explosion in 2014 and the arid and hot summer of 2015 resulted in an unprecedented outbreak over 2 years [26].

### 2.2. Dengue Data

Dengue fever is included on Taiwan’s National Notifiable Infectious Disease Category 2 list. Local physicians are required to report suspected cases within 24 h. Dengue cases were confirmed by serological, nucleotide, or nonstructural protein 1 rapid antigen tests following the Taiwan Centers for Disease Control guidelines. Indigenous dengue cases in 2014 and 2015 were acquired from the Taiwan CDC Open Data Portal (data.cdc.gov.tw (accessed on 15 Dec 2021)). Imported dengue cases were excluded from the analysis because they were irrelevant to local transmission patterns. Taiwan CDC aggregates the dengue case data at the Basic Statistical Area (BSA) level using their residential address. We further summarized the dengue incidence at the second-level dissemination area, roughly equivalent to the village level. BSA is the finest scale of the administrative unit in Taiwan. Our previous study reports detailed information on the administrative units in Taiwan [27]. We used the second-level dissemination area for the analyses to reduce the bias caused by the modifiable area unit problem and maintain sufficient variability in the LULC types. We also excluded mountainous regions (elevations above 300 m) in the northeastern part of KC because of the low vector and residential population density, which made dengue transmission unlikely. The dengue incidence rates were log-transformed in the analysis to reduce a highly skewed distribution (Figure 1a).

### 2.3. Landscape-Level Variables

LULC data were retrieved from the second nationwide land-use investigation conducted by Taiwan’s National Land Surveying and Mapping Center, the Ministry of the Interior [28]. The investigation was launched in 2006 and updated continuously until 2015. The LULC types have been classified through integrating satellite images (SPOT-6 and 7, the spatial resolution is 1.5 m), aerial photos operated by crewless aerial vehicles (the spatial resolution is 25 cm), and ground-based surveys. There are three hierarchical categories for the LULC types: level-1 (9 types), level-2 (43 types), and level-3 (108 types). We used level-3 variables to calculate the percentages of specific LULC types in each spatial unit (the second-level dissemination area) in the model. Table S1 details the cross-reference list of the number and level-3 LULC types. In KC, there are 96 LULC types of level-3 variables. We included 70 LULC types in the analysis after excluding 26 LULC types that cover a meager percentage (<1%) in the second-level dissemination area.

Shannon’s diversity index (SI) is commonly used in ecology to represent the diversity and richness of different species [29]. Recently, this index has been used to analyze the mosquito species diversity and West Nile virus transmission in Costa Rica [30]. Therefore, our study uses this idea to investigate the effect of LULC diversity on the spatial patterns of dengue infection. To capture the diversity of the LULC types per spatial unit, we calculated SI using the following formula:

$$SI_{j}=-\overset{n}{\underset{i=1}{\sum }}p_{ij}lnp_{ij}$$

In this equation, *n* indicates the total number of LULC types, and *pi* is the proportion of a specific LULC type (i) at the *j*th spatial unit in the study area. The minimum SI value is 0, indicating no diversity in the area; however, the higher the SI value, the higher the diversity.

Our preliminary analyses also included climatic variables from a historical weather station summary (10-year averages during the transmission season between May and September). Climate parameters are less critical than LULC factors for determining spatial patterns in the study region; therefore, we removed climate variables from the formal analysis. Finally, we included household density (HD) in the analysis to account for population aggregation. All the data sources in the analysis are listed in Table S2. A multicollinearity assessment was conducted using the variance inflation factor (VIF). The VIF of all the variables was <10; therefore, all the environmental variables were included in the analysis.

### 2.4. Modeling Approaches

XGBoost is a gradient-boosting modeling approach used in various machine learning competitions because of its high performance compared with other tree-based machine learning approaches [31]. In addition, gradient boosted model can be more accurate and interpretable than different modeling approaches on a tabular-style dataset. Therefore, we used XGBoost to analyze the influence of the LULC types and the spatial patterns of dengue infection. Our analysis incorporated an explainable AI approach to enhance our understanding of the model’s output to obtain an accurate and consistent interpretation of the variable influences on dengue incidence [32]. Inconsistent interpretation is a challenge for tree-based or ensemble machine learning approaches. Lundberg et al. proposed the SHAP method for consistent interpretations in 2019 [33,34,35]. SHAP provides detailed explanations as a so-called “black-box machine-learning model” without sacrificing model performance. SHAP is an approach to explain prediction output generated by machine learning models. It helps us to understand the contribution of each variable in the model. The SHAP summary plot can demonstrate global feature importance and local explanation summary, which indicate how each feature affects the dependent variable in the model. The SHAP dependence plot is presented as a scatter plot to show the effect of a feature on the model prediction [36,37].

The formula to calculate the Shapley value is given below:

$$\emptyset_{j}=\underset{S\subseteq F\backslash \left\{j\right\}}{\sum }\frac{\left|S\right|!\left(\left|F\right|-\left|S\right|-1\right)!}{\left|F\right|!}\left[f\left(S\cup \left\{j\right\}\right)-f\left(S\right)\right]$$

where $\emptyset_{j}$ is the Shapley value for sample *j*, *F* the total number of features, $F\backslash \left\{j\right\}$ a set of all possible combinations of features excluding *j*, $f\left(S\right)$ the model prediction with features in *S*, and $f\left(S\cup \left\{j\right\}\right)$ the model prediction with features in *S* plus feature *j*. The Shapley value is the marginal contribution to model prediction averaged over all possible models with different combinations of features.

Figure 1b presents the analytical flow diagram. The sample data were randomly allocated to training (80%) and test (20%) groups. All landscape variables (70 LULC types, SI, and HD) were included in the model training stage, and the final model retained 20 essential variables based on minimizing the loss function. The relative importance of each variable was ranked based on the contribution to the XGBoost model. Ten-fold cross-validation of the training dataset and early stopping approach were performed during the tuning stage to avoid the occurrence of overfitting. The hyperparameters of XGBoost were tuned under different learning rate settings (eta), maximum tree depths, and subsampling rates and validated by the root mean squared error (RMSE). Table S3 details the hyperparameter settings and final optimal parameters. The model performance for the training and test dataset was evaluated by Adj-R squared and RMSE (the formula is given below):

$$\mathrm{RMSE}={\left[\overset{N}{\underset{i=1}{\sum }}{\left({\hat{y}}_{i}-y_{i}\right)}^{2}/N\right]}^{1/2}$$

where N is the total number of observations, ${\hat{y}}_{i}$ the predicted value, and $y_{i}$ the observed value.

SHAP were applied to the output of the XGBoost model to provide better interpretations of landscape-level features and dengue transmission. Variable-specific SHAP values were estimated for each spatial unit to evaluate the positive or negative impacts on dengue transmission. A positive SHAP value indicates that a higher percentage of certain LULC variables in the spatial unit positively associates with dengue incidence. SHAP-dependent plots were generated to demonstrate interpretable associations between the LULC-type variables and dengue transmission.

The predicted dengue incidence rates and residuals derived from the best-fitted XGBoost model in KC were smoothed and visualized using the ordinary kriging method. The risk maps highlight the high-risk areas of dengue transmission in KC. All models were generated using the “XGBoost” and “SHAPforxgboost” packages in R software (version 4.2.2, R Core Team, Vienna, Austria). The risk maps were created using ArcGIS Pro 3.0.3 (ESRI, Redland, CA, USA). The R script used to develop XGBoost/SHAP modeling is available at https://github.com/TMURS/DENGUE_LCLU.

## 3. Results

We identified 933 s-level dissemination areas in KC after removing areas with no data in the past 10 years; 794 samples (80%) were allocated as training data, and 199 samples (20%) were allocated as test data. In 2014 and 2015, 34,782 confirmed dengue cases were reported, and more than 98% of dengue cases (34,329) were successfully aggregated into the second-level dissemination areas using their residential address in our study. The dengue incidence spatial patterns were mainly clustered in the metropolitan region due to the higher population density (Figure 1a). However, small-scale transmission also occurred in suburban and rural neighborhoods.

The XGBoost model included the LULC types (level-3, n = 70), SI, HD, and historical climate variables. However, the historical climatic variables were excluded from the model at the exploratory stage; thus, the model retained 20 important variables based on minimizing the loss function.

**Figure 2:**
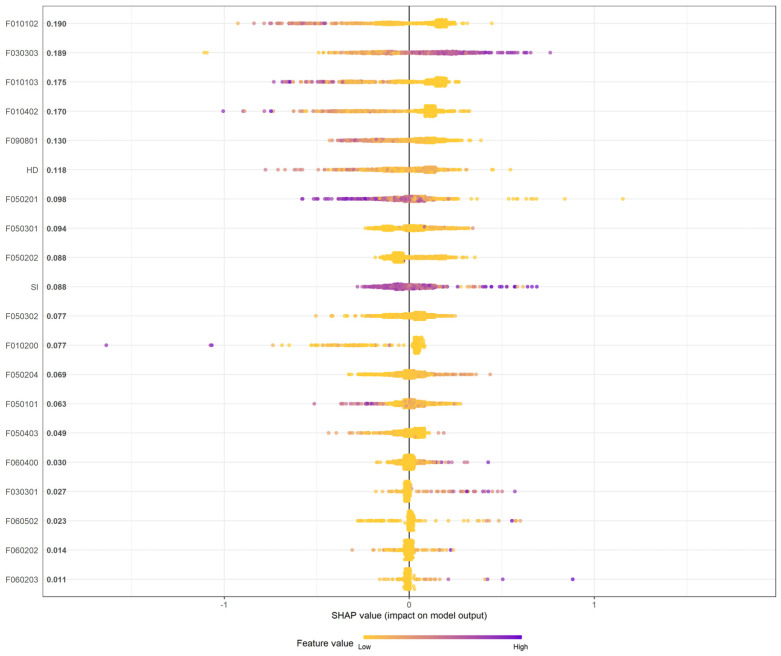
SHAP summary plot. The plot indicated the global variable influences (the relative importance of each variable is ordered) and local explanation (purple color indicates a higher value of corresponding variables and yellow color indicates lower value) on dengue incidence. SHAP, Shapely Additive Explanations.

The global SHAP summary plot retained 20 variables in the output (Figure 2). Among the top 10 important variables, dry crops (F010102) was the most influential variable, showing a protective effect on the spatial patterns of the residences of dengue cases in KC. Other agriculture-related variables (F010103: fruit tree and F010402: aquaculture) demonstrated similar effects on dengue incidence. General roads (F030303) was the second most influential variable. Therefore, areas with more general road coverage were more affected by dengue. Furthermore, unused land (F090801), house density, residential area (F050201), manufacturing (F050301), and industrial areas (F050202) were important variables. SI was also listed as an important factor, indicating that the LULC diversity patterns considerably influenced the spatial distribution of the residences of dengue. Table 1 presents the descriptive statistics of the top ten most important LULC variables.

**Figure 3:**
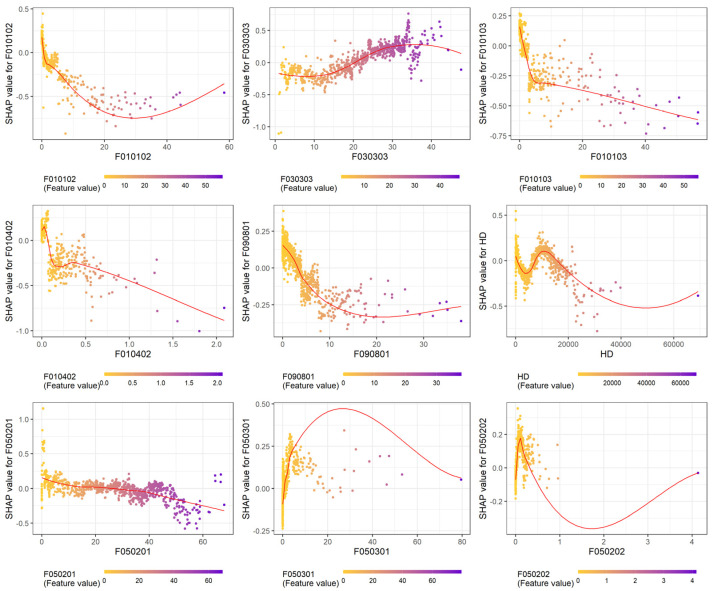
Shapley Additive Explanation (SHAP) dependence plots generated from the EXtreme Gradient Boosting model demonstrate the relationships between the land-use land-cover types and dengue incidence based on the ten most essential variables in Figure 2 and Table 1 (purple color indicates a higher value of corresponding variables and yellow color indicates lower value). The SHAP dependence plot of Shannon’s diversity index is listed in Figure S1 for better typesetting arrangement. A positive SHAP value indicates a higher dengue transmission risk. The plots were smoothed using locally estimated scatterplot smoothing (red curve).

The SHAP-dependent plots demonstrated interpretable associations between the LULC-type variables and dengue incidence (Figure 3 and Figure S1). The results demonstrated that agriculture-related factors (F010102, F010103, and F010402) are inversely associated with dengue incidence. The dengue infection risk increased with >20% general road coverage in KC. An inverted pattern was observed between the residential areas (F050201) and dengue transmission risk, which decreased when the residential area comprised >40% of the total area. HD also had a nonlinear pattern; the incidence rate was higher when the HD was approximately 15,000 but significantly lower when the HD was >20,000. When the second-level dissemination area had a small proportion of unused land (F090801), manufacturing (F050301), and industrial area (F050202), the dengue infection risk was higher. SI had a U-shape association with dengue fever; the infection risk was higher when SI was <1.5 or >2.5.

**Figure 4:**
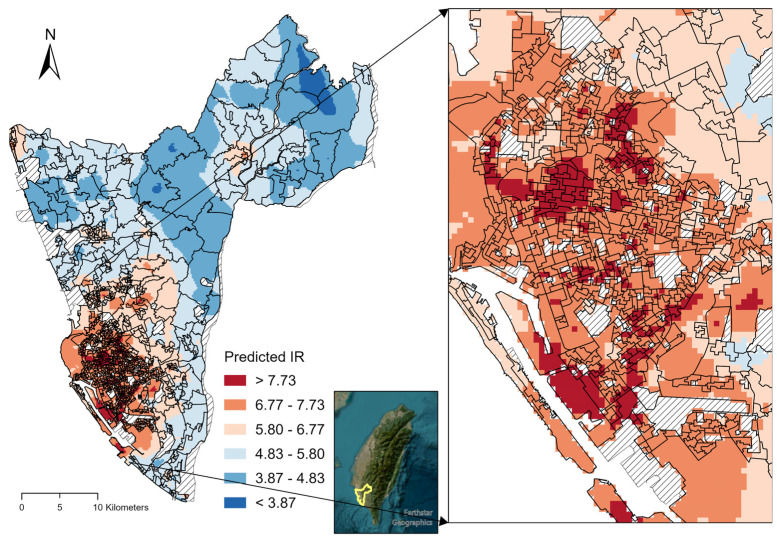
Predicted dengue incidence rates (smoothed by ordinary kriging) in the second-level dissemination areas in Kaohsiung City, Taiwan. The right panel shows the zoomed-in view of the high-risk zones in the metropolitan region.

The dengue infection risk map in KC was visualized using the smoothed predicted incidence rates (Figure 4). The risk map highlights high-risk zones in the metropolitan region and its surrounding neighborhood. Two small hotspots are observed in the northeastern and northwestern corners. In addition, more accurate high-risk zones within metropolitan areas were identified using our model (Figure 4). These areas may be critical zones for dengue vector control and prevention.

**Figure 5:**
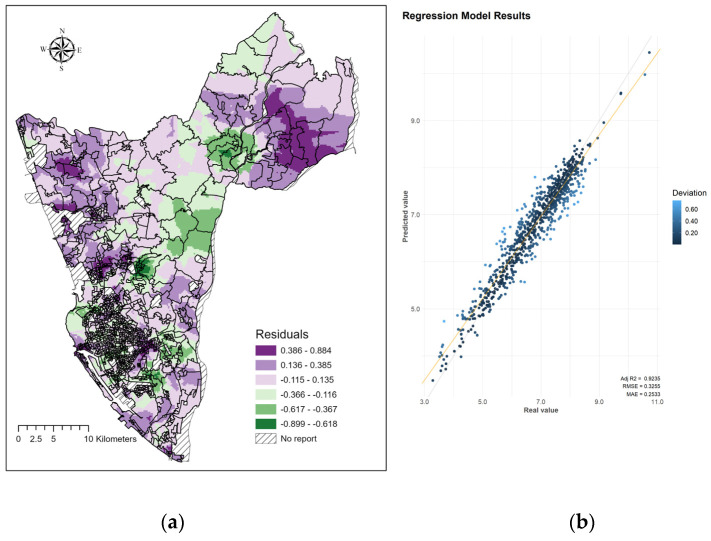
Model validation. (**a**) Spatial patterns of residuals at second-level dissemination areas in Kaohsiung City, Taiwan. (**b**) Comparisons between the predicted and actual dengue incidence rates.

Figure 5 shows the model’s validation results. The residual plots indicate the spatial pattern of the prediction error. Overestimation and underestimation usually occurred in rural and suburban regions, respectively. The XGBoost model performed well with the test data (Adj-R^2^ = 0.92, RMSE = 0.3255). Furthermore, the Adj-R^2^ and RMSE for the test data were 0.78 and 0.6431, respectively, indicating no severe overfitting issue.

## 4. Discussion

This study integrated an explainable AI approach that combined the XGBoost and SHAP methods to analyze fine-scale LULC types and spatial patterns of the residences of reported dengue cases in KC. We found that transportation infrastructure, diversity indexes, and urbanization nonlinearly influenced the spatial patterns of dengue incidence. The XGBoost model also revealed several high-risk zones within metropolitan and suburban areas of KC.

Historically, KC has been a significant dengue hotspot in Taiwan because of the coexistence of *A. aegypti* and *A. albopictus* in the city, and the high population density. However, extreme climatic conditions were the leading cause of the unprecedented KC outbreaks in 2014 and 2015 [17]. Interannual variations in dengue transmission driven by temperatures, rainfall, relative humidity, or regional climate phenomena, such as ENSO and IOD, have been discussed elsewhere [9,11,14,38,39]. Moreover, suitability analyses have demonstrated critical associations between vector ecology, virus propagation, and climatic variations [15,25,40,41]. The abundance of mosquitoes and the extrinsic incubation period of the dengue virus could be shortened by higher temperatures. Precipitation can create more breeding sites [40,41]. Dengue early warning systems have also been developed using climate-based models to forecast the dengue transmission risk [42,43]. Therefore, it is well established that climatic factors are essential for determining the likelihood of outbreaks. However, the spatial distribution of dengue infection can also be affected by other landscapes or anthropogenic factors.

The XGBoost/SHAP approach demonstrated that several LULC variables affect the spatial distribution of residences of dengue cases. In contrast to previous studies, this study used a fine-scale nationwide land-use investigation dataset that incorporated satellite images and field surveys to acquire diverse LULC information. Most previous studies relied on remote sensing data that classified LULC variables into more generalized categories, including forests, wetlands, water bodies, urban areas, croplands, and bare land [44,45,46]. Such a classification may be appropriate for analyzing correlations with dengue transmission or vector ecology at the district or national level. However, a more precise interpretation at a finer spatial resolution may be challenging. In our study, we used more diverse LULC types to analyze their impact on the spatial distribution of the residences of dengue cases. Agriculture-related LULC types showed protective effects on dengue incidence because the agriculture-dominant regions are usually less populated; therefore, transmission is unlikely. Transportation infrastructure (F030303: general roads) was the most important variable in the model, and the risk became significant when the coverage exceeded 20% within a second-level dissemination area. The coverage of general roads is a proxy for population density and anthropogenic activities. Previous studies have shown the importance of road density and distance to roads in dengue transmission in China [47,48]. Our study demonstrates that general roads are accurate, quantifiable targets for the residences of dengue cases.

Dengue fever is an urban-type mosquito borne disease; therefore, the urbanization level plays a critical role in determining the spatial extent of transmission. Our model indicated that residential areas were an important factor affecting the spatial patterns of the residences of dengue cases. In addition, the SHAP dependence plot highlighted the nonlinear association between residential areas and dengue incidence. The nonlinear relationship echoed our previous study, which also identified that dengue incidence was higher when the residential area was approximately 20% in Tainan City [27]. Decreased dengue risk in areas with a high percentage of residential space may be related to a specific type of community. For example, historical dengue hotspots in KC usually occurred in older communities with high contact probability between humans and mosquito vectors. Such communities may mix with unused land or abandoned areas with poor sewer systems and sanitation. In addition, artificial waste containers create breeding sites for mosquito vectors in these areas. However, a high proportion of unused land is usually associated with the low populated area and low transmission.

In contrast, the area with high residential coverage is usually linked to more modern communities or business districts, where dengue transmission is lower than in less developed regions. The U-shaped relationship between SI and the residences of dengue cases also emphasized that a diverse composition of LULC types may either enhance or reduce dengue transmission. This diversity might be linked to human behavior and vector ecology, which is worthy of further analysis.

The XGBoost model performs better than other tree-based models [32], and the model used in this analysis also showed good prediction performance without overfitting. Deep learning (e.g., artificial neural network) approaches also have essential roles in AI; however, such approaches are more appropriate for image recognition and natural language processing [49]. Furthermore, XGBoost is more interpretable than traditional linear regression models. Our recent work also used the combined XGBoost and SHAP approach to evaluate *Plasmodium knowlesi* and landscape-level variables in peninsular Malaysia [50]. The explainable AI approach adopted in this study provides important insights into the complex environmental influences on the spatial distribution of the residences of dengue cases. Furthermore, the high-risk zones predicted in this study (Figure 4) provide more accurate information for prioritizing vector control and dengue prevention strategies. Future research should aim to improve public health by integrating climatic variations and human morbidity to capture the spatial and temporal dynamics of dengue infection.

This study had some limitations. First, the analysis did not include vector abundance and larval indices because the data was unavailable. Many studies have indicated that the landscape level could be an important predictor of mosquito abundance; however, the associations between the vector index and incidence are usually arbitrary [51,52]. A systematic ovitrap surveillance project was implemented in KC in 2016. Therefore, mosquito abundance data should be included in future studies. Second, LULC data from nationwide land-use investigations are only available for Taiwan. However, using more localized datasets, the explainable AI approach can still be applied to different regions using more localized datasets.

Furthermore, the influences of LULC types could not be considered as the cause of dengue transmission. Instead, the LULC types represented the proxy of underlying dengue transmission dynamics driven by the mixture effects of sociodemographic characteristics, human behavior, vector behavior, disease control policy, and climate effects. Lastly, the source of dengue infection could not be identified in the study. The dengue case data were aggregated at the second-level dissemination areas based on the residential address. Therefore, the reported address might not be the exact location of the infection. Wen et al. conducted a study to discuss the spatial and temporal diffusion patterns of dengue in Tainan City, Taiwan. Their result indicated that most noncommuter dengue cases clustered within 100 m, and most commuters clustered within 2–4 km [53]. The results of this study partially supported that the source of dengue transmission might be at a limited distance around the residence for noncommuter dengue cases. The reported dengue cases in the second-level dissemination areas might be associated with the transmission patterns. The study also indicated that a substantial proportion of dengue cases might be infected far away from their home; thus, our conclusion could not be applied to commuter dengue cases. Dengue transmission is intertwined with vector abundance, host density and mobility, and intervention. Our study results only can be interpreted as the associations between LULC factors and the spatial patterns of the residences of reported dengue cases. No causal inference can be made from our results.

Coronavirus disease 2019 (COVID-19) has caused a global pandemic since 2020; however, the impacts of COVID-19 on dengue transmission vary in different regions. Increased dengue transmission has been reported in several countries in Asia and South America, like Thailand, Singapore, and Peru [54,55,56]. The lockdown policy might be an important contributor to enhancing dengue transmission through higher human and vector contact probability, especially for *A. aegypti*. Dengue incidence increased by approximately 37% in Singapore among adults during the COVID-19 pandemic [55]. In addition, the huge proportion of medical resources and public health infrastructures used to deal with COVID-19 during the pandemic period could have reduced the resources for surveillance, intervention, diagnosis, and treatment for dengue and other health outcomes. Rebuilding dengue control and surveillance systems and relocating the intervention resources in the post-pandemic era could be challenges for these countries. Taiwan has reported few indigenous dengue cases after 2016, and no indigenous case has been reported during the pandemic period, throughout 2020–2022. The border control implemented in March 2020 was a crucial policy to limit dengue transmission in Taiwan.

In contrast to other dengue-prevalent countries in Southeastern Asia, international travelers import the dengue virus in spring. This induces subsequent local dengue transmission in Taiwan during summer and fall [57]. The epidemiological characteristic of dengue transmission in Taiwan explained why no indigenous dengue cases were reported during the COVID-19 pandemic. However, the border reopened and quarantine requirements ended in October 2022, bringing a potential challenge of a future dengue epidemic in Taiwan again. In the future, the dengue early warning system should be optimized to integrate climate, LULC types, vector abundance, and human behavioral characteristics to enhance the precision and accuracy of warning signals in space and time. Our study demonstrated a promising machine learning approach to simultaneously balance model performance and interpretability that might be useful for future model development.

## 5. Conclusions

This study integrated explainable machine learning approaches to investigate associations between fine-scale landscape characteristics and spatial patterns of the residences of reported dengue cases in KC, Taiwan. Using the XGBoost and SHAP approaches, we identified nonlinear patterns between diverse LULC types and the spatial patterns of the residences of dengue infection in KC. Furthermore, the predicted high-risk areas within the metropolitan and suburban areas in KC provide essential information for prioritizing dengue interventions. Therefore, explainable AI approaches with XGBoost and SHAP value estimations could enhance our understanding of environmental characteristics and spatial patterns of dengue infection. The information derived from the explainable AI approach is beneficial for resource allocation and modification of dengue control and prevention strategies.

## Figures and Tables

**Table 1:** Descriptive statistics of the ten most influential land-use land-cover types from the SHAP summary.

LULC Types	Mean	Standard Deviation	Max	Min
F010102 (Dry Crops)	2.98%	6.85%	58.31%	0
F030303 (General Roads)	21.10%	10.00%	47.60%	0.91%
F010103 (Fruit Tree)	3.39%	7.74%	56.83%	0
F010402 (Agriculture Storage Facility)	0.10%	0.21%	2.08%	0
F090801 (Unused Land)	4.20%	5.10%	38%	0
House Density	8643.1	7008.6	69,215.2	3.0
F050201 (Residential Area)	26.20%	16.20%	67.90%	0
F050301 (Manufacturing)	1.98%	5.92%	80%	0
F050202 (Industrial Area)	0.06%	0.17%	4.13	0
Shannon’s Diversity Index	1.9	0.4	3.0	0.28

## Data Availability

Dengue case data at the BSA level in this study can be acquired from Taiwan CDC Open Data Portal (data.cdc.gov.tw). The LULC data from the nationwide land use investigation of this study are available on request from the corresponding author.

## Supplementary Materials

The following supporting information can be downloaded at: https://www.mdpi.com/article/10.3390/tropicalmed8040238/s1, Figure S1: Shapley Additive Explanation (SHAP) dependence plots of SI; Table S1: Classifications of third-level land-cover land-use types in Kaohsiung City, Taiwan; Table S2: Sources of dependent and independent variables in the analysis. Table S3: Hyperparameter tuning details for XGBoost modelling.
