# Supplementary material for: An Integrative Explainable Artificial Intelligence Approach to Analyze Fine-Scale Land-Cover and Land-Use Factors Associated with Spatial Distributions of Place of Residence of Reported Dengue Cases
Source: Trop Med Infect Dis. 2023 Apr 20;8(4):238. doi: 10.3390/tropicalmed8040238 (PMC10142856; doi:10.3390/tropicalmed8040238)
Supplement: Supplementary file 1 [file tropicalmed-08-00238-s001.zip › tropicalmed-2275659-supplementary.pdf]

Supplementary Materials: An Integrative Explainable Artificial Intelligence Approach to Analyze Fine-Scale Land-Cover and Land-Use Factors Associated with Spatial Distributions of Place of Residence of Reported Dengue Cases

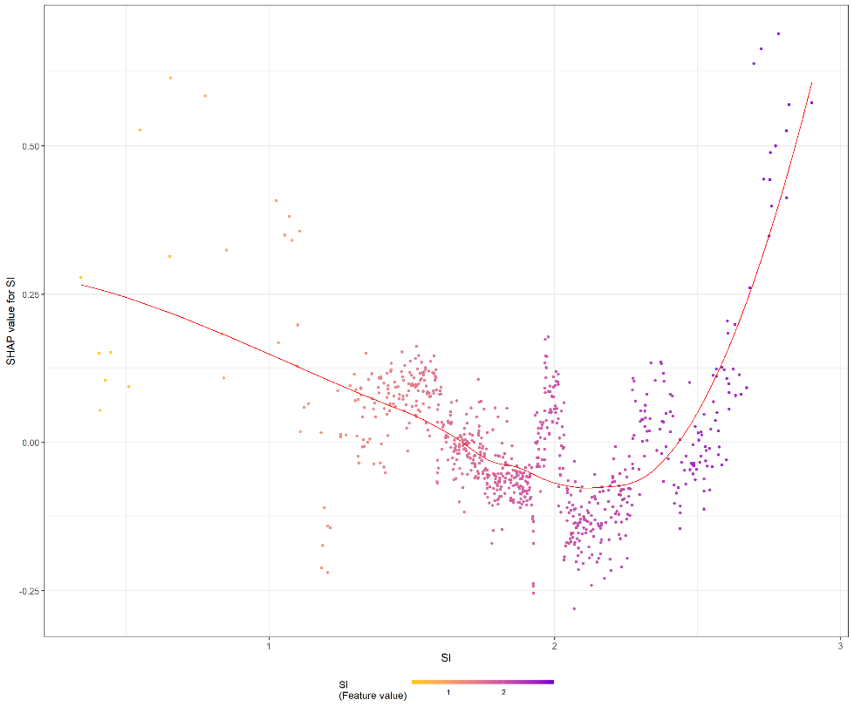

Figure S1. Shapley Additive Explanation (SHAP) dependence plots of SI.

Table S1. Classifications of third-level land-cover land-use types in Kaohsiung City, Taiwan.

No.	Description
F010101	Rice
F010102	Dry crops
F010103	Fruit tree
F010104	Abandoned field
F010200	Aquaculture
F010301	Livestock house
F010302	Pasture
F010401	Greenhouse
F010402	Agriculture Storage facility
F010403	Agricultural product exhibition and sales
F010404	Others
F020101	Natural needle leaf forest
F020102	Natural broad leaf forest
F020103	Natural bamboo forest
F020104	Natural mixed forest
F020201	Artificial needle leaf forest
F020202	Artificial broad leaf forest
F020203	Artificial bamboo forest

F020204	Artificial mixed forest
F020301	Logging site
F020302	Tree Nursery
F020303	Firebreak
F030100	Airport
F030201	General railway
F030202	High speed railway
F030203	Railway related facilities
F030301	Highway
F030302	Expressway
F030303	General road
F030304	Road-related facilities
F030401	Commercial port
F030402	Fishing port
F030403	Exclusive port
F030404	Other port related facilities
F040101	River
F040103	Canal
F040104	Embankment
F040200	Ditch
F040301	Reservoir
F040302	Lake
F040303	Others
F040304	Artificial lake
F040400	Estuary
F040501	Sluice gate
F040502	Pumping station
F040503	Reservoir dam
F040504	Underground water well
F040505	Other facilities
F040600	Flood control road
F050101	Retail and wholesale
F050102	Industry service
F050201	Residential area
F050202	Industrial area
F050203	Commercial area
F050204	Mixture areas
F050301	Manufacturing
F050302	Warehousing
F050401	Religion
F050402	Funeral facilities
F050403	Under construction
F050404	Others
F060100	Government agencies
F060201	Kindergarten
F060202	Primary school
F060203	High school
F060204	University
F060205	Special school
F060300	Health Facilities
F060400	Social welfare facility
F060501	Meteorological stations
F060502	Electric power facilities
F060503	Liquified natural gas terminal
F060504	Water works
F060505	Gas stations
F060600	Environmental protection facility
F070101	Official cultural assets

F070102	General cultural facilities
F070103	Other cultural facilities
F070201	Park / Green area
F070202	Amusement area
F080101	Mine field
F080102	Mining related facilities
F080201	Stone quarry
F080202	Stone quarry related facilities
F090100	Military land
F090200	Wetlands
F090300	Grassland
F090401	Beach
F090402	Collapse
F090403	Reef rock
F090404	Bare open space
F090500	Shrub wasteland
F090600	Disaster place
F090700	Remaining quarry place
F090801	Unused land
F090802	Artificially changing land

Source: Taiwan's National Land Surveying and Mapping Center, the Ministry of the Interior (www.nlsc.gov.tw).

Table S2. Sources of dependent and independent variables in the analysis.

Variables	Types	Sources
Dengue Confirmed Cases	Dependent Variable	Taiwan Centres for Disease Control
Level-3 Land Use and Land Cover Data	Independent Variables	Taiwan's National Land Surveying and Mapping Center, the Ministry of the Interior
Household Density		National Statistics, Taiwan
Climate Parameters		Central Weather Bureau, Taiwan

Sources: Taiwan's National Land Surveying and Mapping Center, the Ministry of the Interior (www.nlsc.gov.tw); National Statistics, Taiwan (www.stat.gov.tw); Central Weather Bureau, Taiwan (www.cwb.gov.tw).

Table S3. Hyperparameter tuning details for XGBoost modelling.

Parameters	Hyperparameter tuning value range	Optimal value
Number of decision trees (nrounds)	50–1000	150
Maximum depth of a tree (max_depth)	1–10	8
Subsample ratio of columns when constructing each tree (colsample_bytree)	0.5–1	0.7778
Learing rate (eta)	0.1–1	0.3
Minimum loss reduction (gamma)	0–10	0
Subsample ratio of the training instances (subsample)	0.1–1	0.6
